# Temporal misalignment of renal sodium transport promotes non-dipping blood pressure phenotypes

**DOI:** 10.1371/journal.pone.0355222

**Published:** 2026-08-13

**Authors:** Anita T. Layton

**Affiliations:** 1 Department of Applied Mathematics, University of Waterloo, Waterloo, Ontario, Canada; 2 Department of Biology, University of Waterloo, Waterloo, Ontario, Canada; 3 Cheriton School of Computer Science, University of Waterloo, Waterloo, Ontario, Canada; 4 School of Pharmacy, University of Waterloo, Waterloo, Ontario, Canada; International University of Health and Welfare, School of Medicine, JAPAN

## Abstract

Non-dipping blood pressure (BP) phenotypes are strongly associated with cardiovascular and renal morbidity, yet the physiological mechanisms governing nocturnal BP dipping remain incompletely understood. Experimental evidence suggests important roles for circadian timing, renal sodium handling, autonomic regulation, and sleep-wake behavior, but their interactions are difficult to isolate experimentally. We developed an integrated circadian-sleep/wake computational model of long-term BP regulation incorporating rhythmic modulation of renal sympathetic nervous activity, vascular tone, renin-angiotensin-aldosterone signaling, tubular sodium transport, and behavioral sleep-wake influences. The model reproduced a physiologically realistic healthy dipper phenotype with robust nocturnal BP reduction and daytime-predominant natriuresis. Component analyses demonstrated that vascular rhythmicity and sleep-wake modulation were dominant determinants of BP dipping, whereas intrinsic tubular sodium transport rhythmicity primarily regulated sodium excretion timing. Progressive reduction of tubular sodium rhythmicity redistributed natriuresis toward the nighttime period with modest effects on BP dipping. In contrast, altering the phase of tubular sodium transport produced marked effects on both natriuresis timing and BP regulation. Delayed tubular sodium transport phases shifted sodium excretion toward the nighttime period and converted the model from a dipper to a non-dipper phenotype despite preservation of rhythmic oscillations in other physiological systems. Sodium loading and enhanced salt-sensitive tubular sodium reabsorption amplified vulnerability when tubular sodium timing was delayed. These simulations suggest that physiological BP dipping emerges from coordinated temporal interactions among cardiovascular, renal, and behavioral regulatory systems. Non-dipping behavior may therefore arise not only from impaired rhythmicity, but also from misalignment among otherwise preserved physiological oscillators, particularly under sodium-loaded and salt-sensitive conditions.

## Introduction

Blood pressure (BP) exhibits pronounced circadian variation under normal physiological conditions, characterized by higher pressures during the daytime active period and a nocturnal decline during sleep. In healthy individuals, nighttime BP typically decreases by approximately 10–20% relative to daytime values, a pattern commonly referred to as the “dipper” phenotype [1–3]. In contrast, attenuation or absence of the normal nocturnal BP reduction, termed “non-dipping,” is strongly associated with adverse cardiovascular and renal outcomes, including left ventricular hypertrophy, stroke, chronic kidney disease progression, and increased mortality [4,5]. Non-dipping phenotypes are particularly prevalent in salt-sensitive hypertension, chronic kidney disease, diabetes, obesity, aging, and sleep disorders, suggesting that abnormalities in circadian BP regulation may represent an important component of cardiovascular and renal pathophysiology [3].

Despite extensive clinical and experimental investigation, the physiological mechanisms responsible for the emergence of non-dipping behavior remain incompletely understood. Multiple interacting systems contribute to circadian BP regulation, including the autonomic nervous system, vascular tone, sleep-wake transitions, hormonal regulation, renal sodium handling, and intrinsic molecular circadian clocks [6,7]. Experimental studies have demonstrated circadian oscillations in renal transporter expression and activity, glomerular filtration, renin-angiotensin-aldosterone signaling, sympathetic activity, and vascular reactivity [8,9]. At the same time, behavioral sleep-state transitions independently alter sympathetic drive, vascular resistance, and sodium handling [10]. Because these systems interact nonlinearly across multiple physiological timescales, isolating their individual and collective contributions to BP dipping experimentally is challenging.

Renal sodium handling is believed to play a particularly important role in the pathogenesis of non-dipping hypertension. Experimental and clinical studies have linked impaired daytime sodium excretion with nocturnal hypertension and enhanced nighttime natriuresis, suggesting that elevated nighttime BP may partially compensate for inadequate sodium excretion during the active period [11,12]. More broadly, kidney-centered interpretations of non-dipping have proposed that abnormalities in pressure natriuresis and sodium balance may represent central physiological drivers of altered circadian BP regulation [13]. However, the extent to which circadian renal transport rhythms, behavioral sleep-wake modulation, and vascular rhythmicity independently contribute to physiological dipping and coordinated natriuresis remains unclear.

Computational modeling provides a powerful framework for dissecting these interacting mechanisms [14,15]. Mathematical models permit selective manipulation of individual physiological pathways while preserving integrated cardiovascular-renal feedback regulation, thereby enabling mechanistic analyses that are difficult or impossible to perform experimentally. Previous modeling studies have investigated circadian influences on renal hemodynamics, sodium transport, and BP regulation, including the effects of circadian transporter oscillations and time-of-day-dependent pharmacological interventions [16–20]. However, the systems-level interactions among renal circadian transport rhythms, sleep-wake modulation, vascular rhythmicity, and salt-sensitive sodium handling in the emergence of dipper and non-dipper phenotypes remain incompletely characterized.

The objective of the present study was therefore to develop an integrated circadian-sleep/wake model of BP regulation capable of investigating the physiological origins of nocturnal BP dipping and non-dipping behavior. The model incorporates rhythmic modulation of renal sodium transport, renal sympathetic nerve activity, vascular tone, renin-angiotensin-aldosterone signaling, and sleep-wake-dependent autonomic regulation. Using this framework, we examined the relative contributions of individual circadian pathways, the importance of phase coordination among physiological rhythms, and the interaction between circadian timing and salt-sensitive sodium handling. The simulations identify coordinated temporal regulation of renal sodium handling and cardiovascular function as a key determinant of physiological BP dipping and suggest that non-dipping may emerge not only from impaired rhythmicity, but also from misalignment among otherwise preserved physiological oscillators.

## Methods

### Baseline blood pressure regulation model

**Fig 1:**
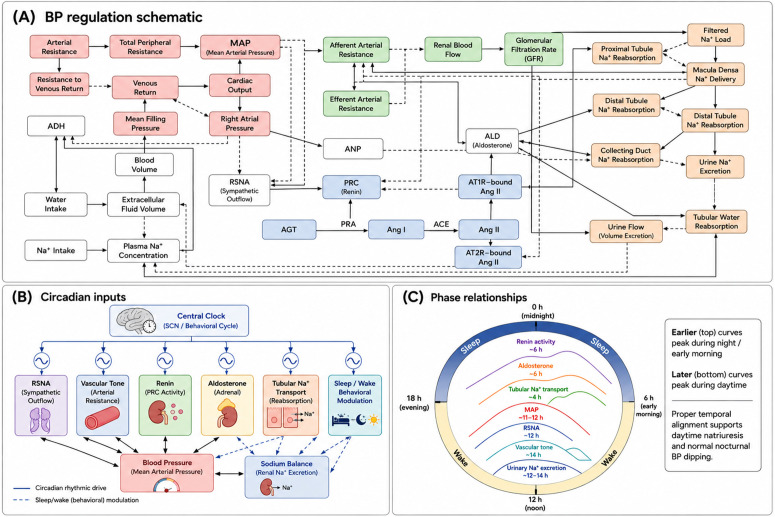
Circadian and sleep-wake modulation of blood pressure regulation. (A) Schematic representation of the integrated blood pressure regulation model, illustrating interactions among cardiovascular function, renal sodium handling, renal sympathetic nervous activity (RSNA), and the renin-angiotensin-aldosterone system (RAAS). Mean arterial pressure (MAP) is determined by the coordinated regulation of vascular resistance, cardiac output, renal hemodynamics, and tubular sodium transport. (B) Extension of the baseline model to include circadian modulation of multiple physiological subsystems, including RSNA, vascular tone, renin secretion, aldosterone signaling, and tubular sodium transport, together with behavioral sleep-wake modulation. Circadian inputs act through partially distinct physiological pathways that converge on BP regulation and sodium balance. (C) Schematic illustration of the relative phase relationships among circadian rhythms incorporated in the model. Sympathetic and vascular rhythms peak during the active phase, whereas renin-aldosterone and tubular sodium transport rhythms exhibit earlier phases. The temporal coordination among these rhythms influences the timing of natriuresis and the emergence of physiological nocturnal BP dipping.

The present study builds upon a previously published mathematical model of long-term BP regulation incorporating coupled cardiovascular, renal, and neurohormonal feedback mechanisms [21,22]. The model is formulated for a premenopausal woman and represents interactions among mean arterial pressure (MAP), renal sodium handling, renal sympathetic nerve activity (RSNA), vascular resistance, and renin-angiotensin-aldosterone system (RAAS) activity. Arterial pressure is determined by the balance between cardiac output and systemic vascular resistance, while renal sodium excretion regulates extracellular fluid volume and long-term BP homeostasis through pressure-natriuresis mechanisms. Fig 1A shows a schematic diagram of the BP regulation model.

The model incorporates dynamic feedback among renal perfusion pressure, tubular sodium reabsorption, renin secretion, angiotensin II signaling, aldosterone-mediated sodium retention, and sympathetic nervous system activation. Renal sodium excretion depends on glomerular filtration and effective tubular sodium reabsorption, which are modulated by both neurohormonal and hemodynamic factors. The resulting framework captures key physiological features of BP regulation, including sodium balance, salt sensitivity, and compensatory renal-cardiovascular feedback interactions.

In the present study, the baseline model was extended to incorporate circadian modulation of RSNA, vascular tone, renin signaling, aldosterone signaling, tubular sodium transport, and sleep-wake-dependent autonomic regulation.

### Circadian and sleep-wake modulation of blood pressure regulation

To investigate the interaction between circadian timing, renal sodium handling, and BP regulation, we extended the baseline integrated BP-kidney model to include explicit circadian and sleep-wake modulation of multiple physiological subsystems. The resulting framework represents BP regulation as a temporally coordinated system in which rhythmic neural, vascular, endocrine, and tubular processes jointly shape daily oscillations in arterial pressure and urinary sodium excretion (see Fig. 1B).

### Circadian modulation

Circadian rhythmicity was incorporated through multiplicative sinusoidal forcing functions applied to selected physiological variables and transport processes. For a generic circadian-modulated quantity *X*, the time-dependent modulation factor was represented as


$$\begin{array}{c} F_{X}(t)=\text{1}+A_{X}\;\text{sin}\left(\frac{\text{2}\pi }{\text{2}\text{4}}\left(t-\phi_{X}\right)\right) \end{array}$$

where $A_{X}$ denotes the rhythm amplitude and $\phi_{X}$ denotes the phase (hours). Time t is expressed in hours on a 24-h clock. In all simulations, the modulation factors were constrained to remain positive.

Five physiological systems were assigned independent circadian rhythms: RSNA, vascular tone, renin secretion, aldosterone signaling, tubular sodium transport. Circadian phases were selected to reproduce physiologically realistic temporal ordering of sympathetic, vascular, endocrine, and tubular sodium transport rhythms reported experimentally (see Fig 1C). Specifically, sympathetic and vascular rhythms were assigned daytime-peaking phases, whereas renin and aldosterone rhythms were assigned early-morning phases consistent with known circadian variation in the renin-angiotensin-aldosterone system. Intrinsic tubular sodium transport rhythms were assigned an early active-phase peak consistent with experimentally observed circadian regulation of renal sodium transporters [16,23–26]. These rhythms were incorporated as follows.

#### RSNA.

Circadian modulation of RSNA was implemented by scaling the effective renal sympathetic drive entering the renal hemodynamic and renin secretion pathways:


$$\begin{array}{c} {\text{RSNA}}_{\text{eff}}(t)={\text{RSNA}}_{\text{0}}F_{\text{RSNA}}(t) \end{array}$$

RSNA influences afferent and efferent arteriolar resistance, tubular sodium reabsorption, and renin release in the underlying BP regulation model. The calibrated RSNA rhythm peaked near midday [23] ($\phi_{\text{RSNA}}$ ~ 12 h) with a modest amplitude ($A_{\text{RSNA}}$ = 0.10).

#### Vascular tone.

Circadian variation in vascular tone was represented through modulation of systemic vascular resistance:


$$\begin{array}{c} R_{\text{vas},\text{eff}}(t)=R_{\text{vas},\text{0}}F_{\text{vas}}(t) \end{array}$$

This rhythm affects total peripheral resistance and therefore contributes directly to daily oscillations in MAP. The vascular rhythm was assigned a slightly larger amplitude ($A_{\text{vas}}\;$= 0.13) and a daytime-peaking phase consistent with known circadian variation in vascular tone and BP [27,28] ($\phi_{\text{vas}}$~ 14 h).

#### Renin secretion.

Circadian modulation of renin secretion was incorporated by scaling plasma renin concentration (PRC) production:


$$\begin{array}{c} {\text{PRC}}_{\text{prod}}(t)={\text{PRC}}_{\text{0}}F_{\text{ren}}(t) \end{array}$$

This rhythm propagates through the RAAS, influencing angiotensin II (Ang II) formation, aldosterone signaling, renal hemodynamics, and sodium reabsorption. The renin rhythm was assigned a larger amplitude ($A_{\text{ren}}$ = 0.20) and an early morning phase [24] ($\phi_{\text{ren}}$ ~ 6 h), consistent with known circadian variation in RAAS activity.

#### Aldosterone signaling.

Circadian aldosterone signaling was represented using an analogous modulation:


$$\begin{array}{c} {\text{ALD}}_{\text{eff}}(t)={\text{ALD}}_{\text{0}}F_{\text{ald}}(t) \end{array}$$

Aldosterone modulates distal tubular and collecting duct sodium transport in the baseline model. The aldosterone rhythm was assigned the same phase and amplitude as the renin rhythm ($A_{\text{ald}}$ = 0.20), ($\phi_{\text{ald}}$ ~ 6 h) [29].

#### Tubular sodium transport.

To represent intrinsic circadian regulation of renal epithelial sodium transport, a separate rhythmic modulation was applied directly to tubular sodium reabsorption processes:


$$\begin{array}{c} T_{\text{Na},\text{eff}}(t)=T_{\text{Na},\text{0}}F_{\text{TNa}}(t) \end{array}$$

This modulation acts independently of hormonal regulation and represents local renal clock influences on epithelial transport. The tubular sodium transport rhythm was assigned a smaller amplitude ($A_{\text{TNa}}$ = 0.06) and an earlier phase [25] ($\phi_{\text{TNa}}$ ~ 4 h), reflecting experimentally observed anticipatory tubular transport rhythms preceding the active period.

The phases and amplitudes of the newly introduced circadian modulation functions were selected based on available experimental measurements of circadian physiology where possible. GFR timing was constrained by reported diurnal variations in renal hemodynamics, while renin and aldosterone rhythms were based on human endocrine studies. Circadian modulation of tubular sodium transport was guided by experimental evidence for clock-dependent regulation of major renal sodium transporters, although direct measurements of whole-kidney transport rhythmicity in humans are not available. Likewise, sleep–wake modulation of renal sympathetic nerve activity and vascular tone was based on established physiological reductions in sympathetic activity and vascular resistance during normal sleep. Where direct quantitative measurements were unavailable, parameter values were calibrated to reproduce a physiologically realistic healthy dipper phenotype and subsequently evaluated through sensitivity analysis.

### Sleep-wake modulation

In addition to intrinsic circadian oscillations, we incorporated a separate sleep-wake modulation intended to represent behavioral and state-dependent influences not captured by the endogenous circadian clock alone. These include posture, physical activity, feeding state, autonomic tone, and behavioral renal responses associated with sleep and wakefulness.

Sleep-wake modulation was implemented using a smoothed rectangular function defining a nocturnal interval centered on the sleep phase:


$$\begin{array}{c} S_{X}(t)=\text{1}-A_{\text{sleep},X}G(t) \end{array}$$

where $A_{\text{sleep}}$ denotes the sleep-associated suppression amplitude. The sleep-wake gating function G(t) was defined to take values near 1 during the designated sleep interval (approximately 22:00–06:00) and near 0 during wakefulness, thereby representing behavioral state-dependent modulation of cardiovascular and renal function.

Sleep-wake modulation acted on two primary physiological pathways. First, sleep-associated reductions in sympathetic and vascular drive were incorporated by scaling both RSNA and vascular tone during the sleep interval. Experimental studies have demonstrated reduced renal sympathetic nerve activity during sleep compared with wakefulness [10,30]. Normal sleep is associated with reduced sympathetic activation, lower arterial pressure, and reduced vascular tone relative to wakefulness [10,31].

Specifically, sleep-wake modulation was incorporated multiplicatively, such that the total effective renal sympathetic activity and vascular tone were given by the products of the circadian modulation terms and the corresponding sleep-wake modulation factors:


$$\begin{array}{c} {\text{RSNA}}_{\text{total}}(t)={\text{RSNA}}_{\text{eff}}(t)S_{\text{RSNA}}(t) \end{array}$$

$$\begin{array}{c} R_{\text{vas},\text{total}}(t)=R_{\text{vas},\text{eff}}(t)S_{\text{vas}}(t) \end{array}$$

During the sleep interval, the sleep-wake modulation factors decreased below unity, representing reduced sympathetic and vascular drive during sleep. The sleep-associated suppression amplitudes $A_{\text{sleep},\text{RSNA}}$ and $A_{\text{sleep},\text{vas}}$ are taken to be 0.1 and 0.2, respectively.

Second, a sleep-associated reduction in effective tubular sodium reabsorption (“sleep natriuresis relief”) was incorporated to facilitate physiologic nocturnal sodium handling. This mechanism was motivated by experimental and clinical observations linking circadian sodium excretion patterns with nocturnal BP dipping, as well as studies suggesting that impaired daytime natriuresis contributes to nocturnal hypertension and non-dipping phenotypes [12,32], and is represented by


$$\begin{array}{c} T_{\text{Na},\text{total}}(t)=T_{\text{Na},\text{eff}}(t)\left(\text{1}-\eta_{\text{sleep}}G(t)\right) \end{array}$$

where $\eta_{\text{sleep}}$ controls the degree of nocturnal reduction in tubular sodium reabsorption, and is taken to be 0.25. Physiologically, this mechanism represents reduced nocturnal sodium avidity and facilitates nighttime sodium excretion.

### Salt-sensitive tubular sodium reabsorption

To investigate interactions between circadian timing and salt-sensitive renal physiology, additional simulations incorporated enhanced tubular sodium reabsorptive drive representing a salt-sensitive phenotype. Salt sensitivity was modeled phenomenologically through a multiplicative scaling factor applied to effective tubular sodium reabsorption. Specifically, baseline tubular sodium transport activity was multiplied by a salt-sensitive tubular sodium reabsorption multiplier, $S_{\text{SS}}$, such that


$$\begin{array}{c} T_{\text{Na},\text{e}\text{ff}}(t)={S_{\text{SS}}T}_{\text{Na},\text{base}}(t) \end{array}$$

where $T_{\text{Na},\text{base}}(t)$denotes the baseline circadian tubular sodium transport term and $T_{\text{Na},\text{eff}}(t)$ represents the resulting effective tubular sodium reabsorptive activity. Values of $S_{\text{SS}}>\text{1}$ therefore correspond to enhanced sodium retention and reduced pressure-natriuresis efficiency.

This phenomenological representation was intended to capture the integrated physiological consequences of salt-sensitive renal sodium handling, including enhanced tubular sodium reabsorption, impaired natriuretic responsiveness, and increased susceptibility to sodium-dependent BP elevation, without explicitly modeling specific molecular transporter abnormalities. Simulations were performed across a range of salt-sensitive reabsorption multipliers in combination with varying sodium intake levels and tubular sodium transport phase relationships.

### Calibration of the baseline dipper phenotype

Model parameters governing circadian modulation amplitudes and sleep-wake effects were calibrated to reproduce a physiologically realistic healthy dipper phenotype (Table 1). Calibration targets included mean arterial pressure near 90 mmHg, nocturnal BP reduction within the normal dipper range (~10–20%), daytime-predominant natriuresis, physiologic circadian timing of MAP and GFR rhythms, and stable 24-h sodium balance. A sleep-associated natriuresis relief mechanism was additionally introduced to avoid unrealistically complete suppression of nighttime sodium excretion while preserving robust nocturnal BP dipping.

**Table 1:** Circadian and sleep-wake modulation parameters used in the calibrated baseline model. Circadian modulation terms were represented using sinusoidal functions with 24-h periodicity. The sleep interval was defined approximately from 22:00 to 06:00. Amplitudes are dimensionless fractional modulation strengths relative to baseline values.

Physiological subsystem	Symbol	Amplitude	Peak (h)	Period (h)	Functional effect
Renal sympathetic nervous activity	$F_{\text{RSNA}}(t)$	0.10	12	24	Modulates renal sympathetic drive, renal vascular resistance, renin secretion, and tubular sodium reabsorption
Vascular tone	$F_{\text{vas}}(t)$	0.13	14	24	Modulates systemic vascular resistance
Renin secretion	$F_{\text{ren}}(t)$	0.20	6	24	Modulates plasma renin production
Aldosterone signaling	$F_{\text{ald}}(t)$	0.20	6	24	Modulates distal tubular sodium transport
Tubular sodium transport	$F_{\text{TNa}}(t)$	0.06	4	24	Modulates intrinsic tubular sodium reabsorption
Sleep-associated RSNA suppression	$S_{\text{RSNA}}(t)$	0.10	Sleep interval	—	Reduces effective RSNA during sleep
Sleep-associated vascular suppression	$S_{\text{vas}}(t)$	0.20	Sleep interval	—	Reduces effective vascular tone during sleep
Sleep natriuresis relief	$\eta_{\text{sleep}}$	0.25	Sleep interval	—	Reduces effective tubular sodium reabsorption during sleep

### Rhythm component and phase-shift studies

To investigate the relative contributions of individual circadian pathways to BP dipping and natriuresis, selective rhythm component analyses were performed in which the amplitude of a specific rhythmic modulation was reduced or set to zero while preserving all remaining model components. Additional studies varied the amplitude and phase of intrinsic tubular sodium transport rhythmicity to examine the importance of circadian timing alignment among renal and cardiovascular processes. Separate simulations additionally examined interactions between sodium loading, salt-sensitive tubular sodium reabsorption, and tubular sodium transport phase alignment.

### Local parameter sensitivity analysis

To assess the robustness of the model predictions, a local one-at-a-time parameter sensitivity analysis was performed. Three parameters directly related to the mechanisms investigated in this study were independently perturbed by ±5% from their calibrated baseline values: (i) the amplitude of sleep–wake modulation of renal sympathetic nerve activity and vascular tone, applied as a common multiplicative scaling factor; (ii) the magnitude of the sleep-associated tubular sodium reabsorption relief term; and (iii) the salt-sensitive tubular sodium reabsorption multiplier. All other model parameters and simulation conditions were held fixed.

For each perturbed parameter set, the model was simulated to its periodic steady state, and the resulting mean arterial pressure (MAP), MAP oscillatory amplitude, and percent nocturnal BP dip were computed over the final 24 h. Changes in these quantities were expressed relative to the calibrated baseline solution as percentage differences, providing a measure of the local sensitivity of model predictions to modest parameter uncertainty.

## Results

### Baseline calibrated healthy dipper phenotype

**Fig 2:**
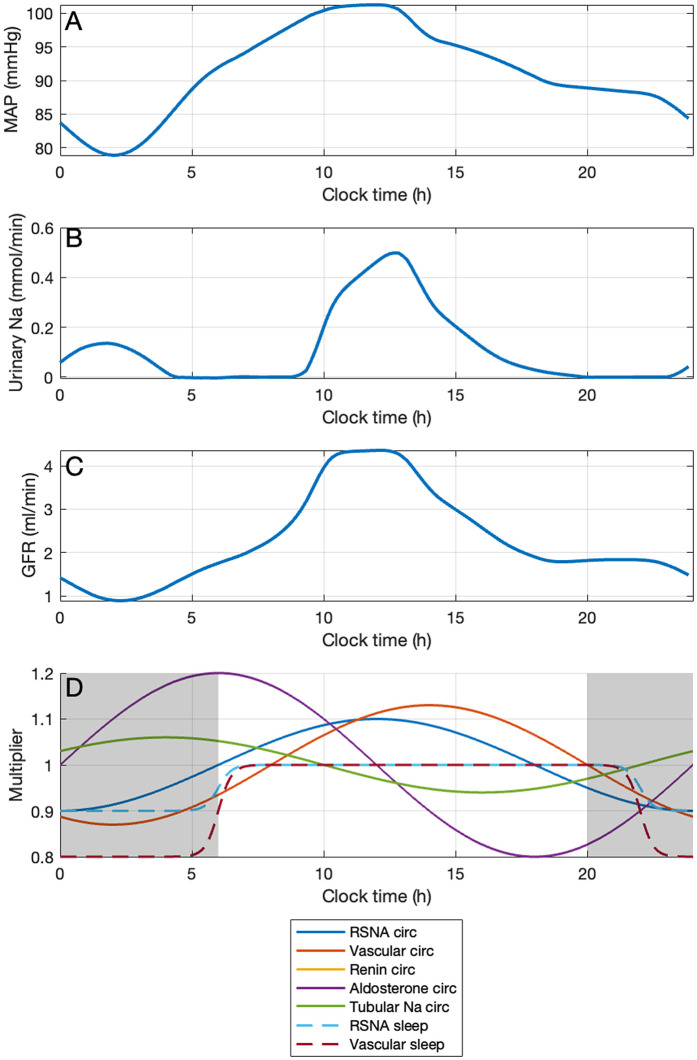
Calibrated healthy dipper baseline phenotype. Simulated 24-h profiles for the calibrated baseline model exhibiting a physiologically realistic dipper phenotype. (A) Mean arterial pressure (MAP) displays a normal nocturnal decline during the sleep interval, with a nighttime reduction of approximately 10–12% relative to daytime values. (B) Urinary sodium excretion exhibits predominantly daytime natriuresis, with reduced sodium excretion during the sleep interval while maintaining overall 24-h sodium balance. (C) Glomerular filtration rate (GFR) exhibits circadian variation with a daytime-predominant profile broadly aligned with the active phase. (D) Circadian and sleep-wake modulation factors incorporated in the model, including renal sympathetic nervous activity (RSNA), vascular tone, renin-aldosterone signaling, tubular sodium transport, and sleep-associated modulation. Shaded regions indicate the designated sleep interval (approximately 22:00–06:00).

The integrated circadian-sleep/wake model was first calibrated to reproduce a physiologically realistic healthy dipper phenotype (Fig 2, Table 2). Under baseline conditions, the model generated a mean MAP of 91.2 mmHg with a 24-h MAP oscillation amplitude of 11.2 mmHg. Simulated daytime and nighttime MAP values were 94.9 and 84.1 mmHg, respectively, corresponding to an 11.4% nocturnal BP reduction, consistent with a normal dipper phenotype reported in ambulatory BP monitoring studies [33,34].

**Table 2:** Baseline calibrated dipper phenotype.

MAP mean (mmHg)	91.2275
MAP amplitude (mmHg)	11.1849
MAP day (mmHg)	94.8798
MAP night (mmHg)	84.0720
Percent dip (%)	11.3910
Dipping status	Dipper
MAP peak/trough	11.83 / 2.00 h
UNa peak/trough	12.67 / 5.83 h
GFR peak/trough	12.17 / 2.33 h
24h UNa (mmol/day)	160.9086
Night/day UNa ratio	0.1723

The simulated MAP waveform exhibited a characteristic daytime rise and nocturnal decline, with peak MAP occurring near midday and trough MAP during the early sleep period (Fig 2A). The model also reproduced coordinated circadian variation in renal function and sodium handling. Glomerular filtration rate (GFR) peaked during the daytime active period and declined during sleep (Fig 2C), while urinary sodium excretion exhibited strongly daytime-predominant natriuresis with markedly reduced nighttime sodium excretion (Fig 2B). The resulting night/day urinary sodium excretion ratio was 0.17, indicating that the majority of sodium excretion occurred during the active phase, consistent with experimental observations in healthy dipper subjects [11,33,35].

To avoid unrealistically complete suppression of nocturnal sodium excretion, the model incorporated a sleep-associated natriuresis relief mechanism that partially reduced effective tubular sodium reabsorption during the sleep interval. This mechanism preserved limited nighttime sodium excretion while maintaining robust nocturnal BP dipping. Under these calibrated conditions, the model achieved stable sodium balance over the 24-h cycle with a total daily urinary sodium excretion of approximately 161 mmol/day, consistent with moderate sodium intake conditions in healthy adults [11].

Together, these results demonstrate that the integrated model reproduces key physiological characteristics of a healthy circadian BP phenotype, including robust nocturnal BP dipping, coordinated daytime natriuresis, and physiologically aligned cardiovascular and renal circadian rhythms.

### Circadian component analysis

**Fig 3:**
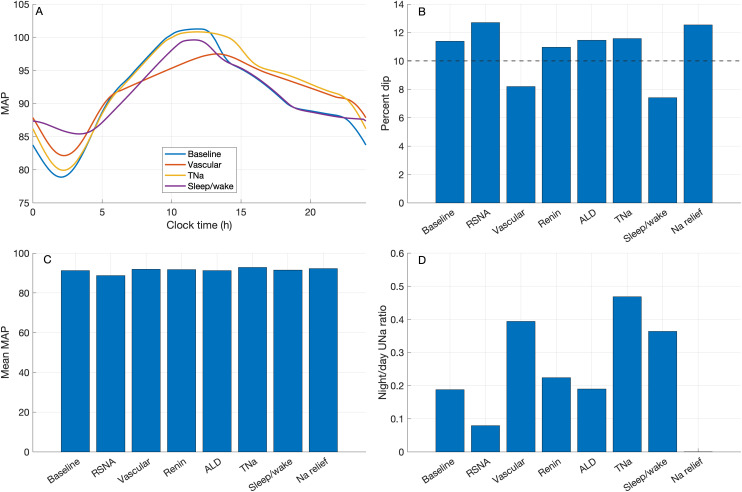
Rhythm component analysis. Selective removal of individual circadian and sleep-wake modulation pathways reveals their contributions to blood pressure dipping and sodium handling. (A) Simulated 24-h mean arterial pressure (MAP) profiles for the baseline model and selected component-removal conditions. Removal of vascular rhythmicity or sleep-wake modulation markedly attenuated nocturnal BP dipping, whereas removal of renin or aldosterone rhythmicity produced comparatively modest effects. (B) Percent nocturnal BP dip across all cases. The dashed horizontal line indicates the conventional 10% threshold separating dipper and non-dipper phenotypes. (C) Mean MAP for each case. Removal of tubular sodium rhythmicity increased mean MAP despite preserving a dipper phenotype. (D) Night/day urinary sodium excretion ratio. Removal of tubular sodium rhythmicity substantially increased nighttime sodium excretion, indicating disruption of physiologic daytime-predominant natriuresis. Together, these simulations demonstrate that vascular and sleep-wake modulation are dominant determinants of BP dipping, whereas intrinsic tubular sodium rhythmicity primarily regulates the temporal distribution of sodium excretion. In panels B-D, xtick labels indicate the components that were removed (except for “Baseline”). ALD, aldosterone; T_Na_, tubular Na^+^ transport; Na relief, sleep natriuresis relief.

To determine the relative contributions of individual circadian and sleep-wake regulatory pathways to physiological BP dipping, selective component analyses were performed in which individual rhythmic inputs were removed while all remaining model components were preserved (Fig 3, Table 3).

**Table 3:** Rhythm component analysis summary. Individual circadian or sleep-wake modulation pathways were removed (labelled under “Case”) while preserving other components. MAP, in mmHg; 24-hour urinary Na^+^ excretion, UNa 24h, given in mmol/day. T_Na_, tubular Na^+^ transport; Na relief, sleep natriuresis relief. Vascular rhythmicity and sleep-wake modulation are dominant determinants of dipping, while tubular Na timing primarily affects sodium excretion timing.

Case	MAP mean	MAP amp	MAP day	MAP night	% dip	UNa 24h	Night/day ratio	Status
Baseline	91.2	11.2	94.9	84.1	11.4	161	0.19	Dipper
RSNA	88.7	12.0	92.6	80.9	12.6	118	0.082	Dipper
Vascular	91.9	7.67	94.5	86.8	8.17	162	0.41	Non-dipper
Renin	91.7	10.8	95.2	84.8	11.0	159	0.23	Dipper
ALD	91.2	11.2	94.9	84.0	11.5	156	0.19	Dipper
T_Na_	92.8	10.5	96.6	85.4	11.6	163	0.48	Dipper
Sleep/wake	91.5	7.10	93.8	86.8	7.41	160	0.364	Non-dipper
Na relief	92.1	12.9	96.3	84.0	12.7	214	4.9E-5	Dipper

Circadian rhythms in RSNA, vascular tone, renin secretion, aldosterone signaling, tubular sodium transport, as well as the two sleep/wake modulation pathways were investigated. Among these, removal of circadian vascular modulation substantially attenuated nocturnal BP dipping, reducing the percent dip from 11.4% to 8.2% and shifting the model from a dipper to a non-dipper phenotype. MAP rhythmic amplitude was similarly reduced. These results are consistent with experimental and clinical evidence demonstrating an important role for circadian vascular function and endothelial regulation in generating physiological BP rhythms. Thus, vascular rhythmicity represented the dominant intrinsic circadian determinant of BP dipping within the calibrated model framework.

Removal of sleep-wake modulation also produced a non-dipper phenotype, reducing nocturnal BP dipping to 7.4%. In this condition, endogenous circadian oscillators remained intact, but behavioral sleep-associated suppression of sympathetic and vascular drive was absent. Together, these results suggest that physiological nocturnal BP reduction emerges from coordinated interactions between intrinsic circadian rhythms and behavioral sleep-state modulation. Experimental studies similarly demonstrate reduced sympathetic activity, vascular tone, and arterial pressure during normal sleep [10,30,36].

In contrast, removal of renin or aldosterone rhythmicity produced comparatively modest effects on BP dipping. Elimination of circadian renin modulation reduced nocturnal BP dipping only slightly, from 11.4% to 11.0%, while removal of aldosterone rhythmicity had minimal impact on the dipper phenotype. These findings suggest that under baseline physiological conditions, circadian oscillations in the RAAS contribute less strongly to short-term BP dipping than vascular or sleep-wake pathways.

Removal of intrinsic tubular sodium transport rhythmicity produced a distinct phenotype. Although nocturnal BP dipping remained preserved (11.6%), mean MAP increased and the temporal distribution of natriuresis changed substantially. Specifically, the night/day urinary sodium excretion ratio increased from 0.19 to 0.48, indicating marked redistribution of sodium excretion toward the nighttime period. These simulations suggest that intrinsic tubular sodium transport rhythms primarily regulate the timing of sodium excretion rather than serving as dominant drivers of BP dipping itself.

Removal of the sleep natriuresis relief mechanism produced the opposite behavior. In this condition, nocturnal BP dipping increased to 12.7%, but nighttime urinary sodium excretion became nearly absent, with the night/day urinary sodium excretion ratio approaching zero. Thus, the sleep natriuresis relief pathway was important for maintaining physiologically realistic nocturnal sodium handling despite robust BP dipping.

Overall, the integrated model containing all circadian and sleep-wake pathways generated the most physiologically coordinated phenotype, simultaneously reproducing robust nocturnal BP dipping and physiologic daytime-predominant natriuresis. Together, these component analyses demonstrate that vascular rhythmicity and sleep-wake modulation are dominant determinants of BP dipping, whereas intrinsic tubular sodium rhythmicity primarily governs the temporal distribution of sodium excretion.

### Effect of tubular sodium transport rhythm amplitude on BP dipping and natriuresis

**Fig 4:**
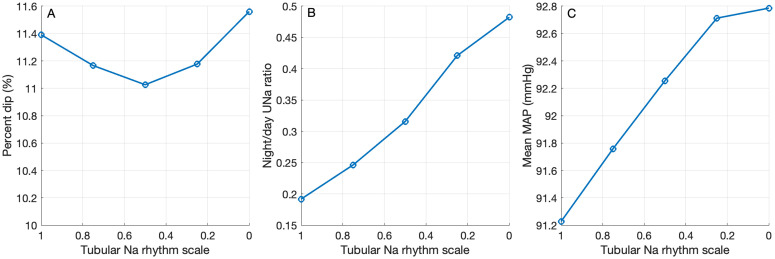
Effect of tubular sodium transport rhythm amplitude on blood pressure dipping and natriuresis. Progressive reduction of the intrinsic tubular sodium transport circadian rhythm amplitude altered sodium excretion timing while producing comparatively modest changes in nocturnal BP dipping. (A) Percent nocturnal BP dip remained within the dipper range across the amplitude sweep, despite a gradual reduction in MAP rhythmicity. (B) The night/day urinary sodium excretion ratio increased progressively as tubular sodium rhythmicity was reduced, indicating a shift toward greater nighttime sodium excretion. (C) Mean MAP increased modestly with decreasing tubular sodium rhythmicity. Together, these simulations suggest that intrinsic tubular sodium transport rhythms primarily regulate the temporal distribution of natriuresis rather than serving as the dominant determinant of BP dipping in the calibrated healthy baseline phenotype.

To investigate the specific contribution of intrinsic tubular sodium transport rhythmicity to circadian BP regulation, the amplitude of the tubular sodium transport rhythm was progressively reduced while all other circadian and sleep-wake modulation pathways were preserved (Fig 4, Table 4).

**Table 4:** Effect of tubular sodium transport rhythm amplitude on blood pressure dipping and sodium handling. Summary of simulations in which the amplitude of the intrinsic tubular sodium transport circadian rhythm was progressively reduced while all other circadian and sleep-wake modulation pathways were preserved. Decreasing tubular sodium rhythmicity modestly increased mean arterial pressure and altered the temporal distribution of urinary sodium excretion, as reflected by increases in the night/day urinary sodium excretion ratio. In contrast, the magnitude of nocturnal BP dipping remained comparatively preserved across the amplitude sweep, indicating that intrinsic tubular sodium rhythmicity exerts a stronger influence on natriuresis timing than on BP dipping in the calibrated healthy baseline phenotype.

T_Na_ rhythm scale	$A_{𝐓𝐍𝐚}$	MAP mean	MAP amp	Percent dip	UNa 24h	Night/day ratio	Status
1	0.06	91.2	11.2	11.4	161	0.19	Dipper
0.75	0.045	91.8	10.9	11.2	164	0.25	Dipper
0.5	0.03	92.3	10.6	11.0	166	0.32	Dipper
0.25	0.015	92.7	10.5	11.2	163	0.42	Dipper
0	0.0	92.8	10.5	11.6	163	0.48	Dipper

Reducing tubular sodium transport rhythmicity produced comparatively modest effects on nocturnal BP dipping. Across the full amplitude sweep, the model remained within the dipper range, with nocturnal BP reduction varying only slightly from 11.4% in the baseline condition to approximately 11.6% in the absence of tubular sodium rhythmicity. MAP rhythmic amplitude decreased modestly as tubular sodium rhythmicity was reduced, while mean MAP increased progressively from 91.2 to 92.8 mmHg.

In contrast, the temporal distribution of sodium excretion was strongly affected by tubular sodium rhythm amplitude. Progressive reduction of tubular sodium rhythmicity increased the night/day urinary sodium excretion ratio from 0.19 under baseline conditions to 0.48 when tubular sodium rhythmicity was completely removed, indicating substantial redistribution of natriuresis toward the nighttime period. Representative simulations demonstrated delayed and broadened urinary sodium excretion profiles as tubular sodium rhythmicity weakened, despite preservation of overall BP dipping.

Interestingly, total 24-h urinary sodium excretion remained comparatively stable across the amplitude sweep, indicating that reduction of tubular sodium rhythmicity primarily altered the timing rather than the total magnitude of sodium excretion. These findings suggest that intrinsic tubular sodium transport rhythms are important determinants of coordinated daytime-predominant natriuresis but are not, by themselves, dominant generators of the dipper phenotype in the calibrated healthy baseline state.

### Interaction between dietary sodium intake and tubular sodium transport rhythmicity

**Fig 5:**
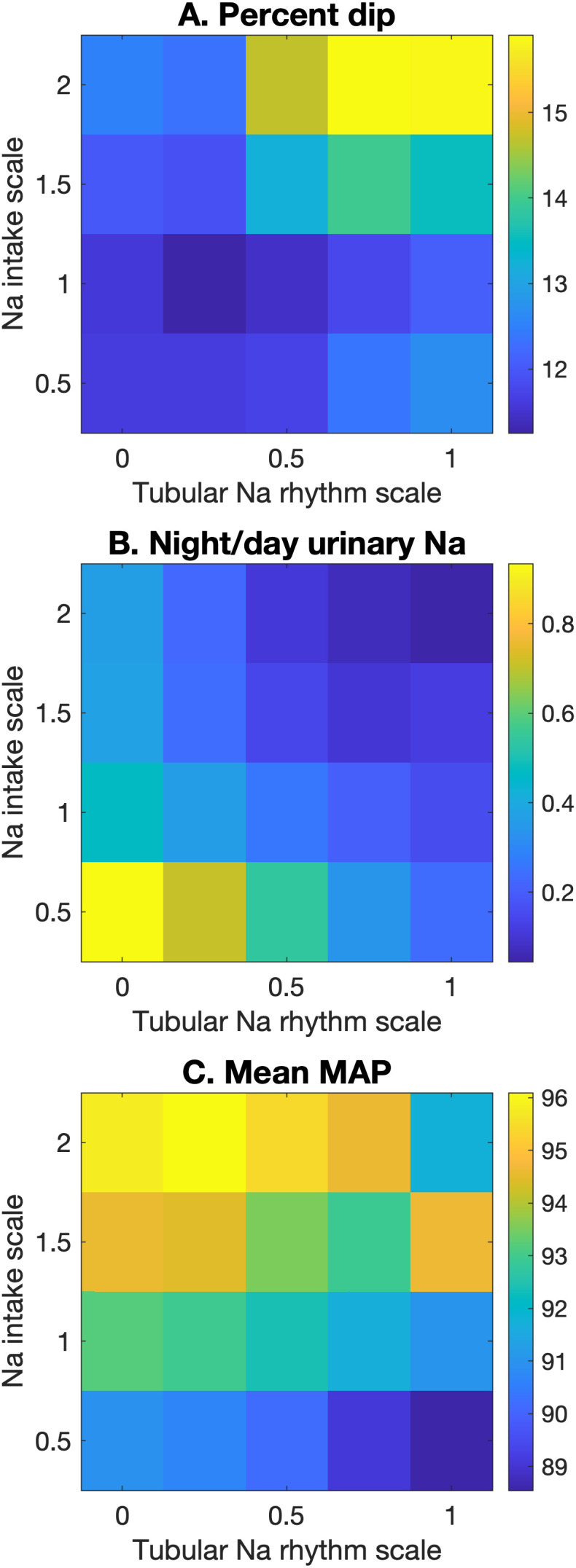
Interaction between dietary sodium intake and tubular sodium transport rhythmicity. Heatmaps illustrating the combined effects of dietary sodium intake and intrinsic tubular sodium transport circadian rhythmicity on blood pressure regulation and sodium handling. (A) Percent nocturnal BP dip increased with stronger tubular sodium rhythmicity, particularly under high sodium intake conditions. (B) The night/day urinary sodium excretion ratio decreased progressively with increasing tubular sodium rhythmicity, indicating enhanced daytime-predominant natriuresis. (C) Mean arterial pressure (MAP) increased with dietary sodium loading but was attenuated by stronger tubular sodium rhythmicity. Together, these simulations demonstrate that circadian coordination of tubular sodium transport becomes increasingly important for maintaining physiological sodium excretion patterns and BP regulation during sodium loading.

To examine how tubular sodium transport rhythmicity interacts with sodium loading, simulations were performed across combinations of dietary sodium intake and tubular sodium transport rhythm amplitude (Fig 5, Table 5).

**Table 5:** Interaction between dietary sodium intake and tubular sodium transport rhythmicity. Summary of simulations examining the combined effects of dietary sodium intake and intrinsic tubular sodium transport circadian rhythmicity on blood pressure regulation and sodium handling. Increasing dietary sodium intake amplified the physiological impact of tubular sodium transport rhythmicity. At higher sodium intake levels, stronger tubular sodium rhythmicity was associated with lower mean arterial pressure, enhanced nocturnal BP dipping, and greater daytime-predominant natriuresis, reflected by reduced night/day urinary sodium excretion ratios. These results suggest that circadian coordination of renal sodium transport becomes increasingly important for maintaining physiological BP regulation during sodium loading.

Na intake scale	T_Na_ rhythm scale	MAP mean	MAP amp	Percent dip	UNa 24h	Night/day ratio	Status
0.5	0.0	91.0	10.3	11.6	80.3	0.93	Dipper
1.0	0.0	93.2	10.4	11.6	163	0.47	Dipper
1.5	0.0	94.6	10.7	12.0	247	0.38	Dipper
2.0	0.0	95.8	11.1	12.5	333	0.37	Dipper
0.5	0.25	90.7	10.4	11.6	80.4	0.71	Dipper
1.0	0.25	93.0	10.4	11.3	166	0.36	Dipper
1.5	0.25	94.4	10.7	11.9	246	0.24	Dipper
2.0	0.25	96.1	11.0	12.3	330	0.23	Dipper
0.5	0.5	90.2	10.6	11.7	78.3	0.54	Dipper
1.0	0.5	92.4	10.8	11.5	163	0.26	Dipper
1.5	0.5	93.5	11.6	13.2	245	0.15	Dipper
2.0	0.5	95.4	12.1	14.7	328	0.11	Dipper
0.5	0.75	89.1	10.9	12.4	78.1	0.34	Dipper
1.0	0.75	91.7	11.3	11.8	148	0.20	Dipper
1.5	0.75	92.9	12.0	14.0	252	0.10	Dipper
2.0	0.75	94.6	13.0	15.9	329	0.071	Dipper
0.5	1.0	88.5	10.9	12.7	84.9	0.24	Dipper
1.0	1.0	91.0	11.6	12.1	140	0.16	Dipper
1.5	1.0	94.6	13.2	13.5	337	0.12	Dipper
2.0	1.0	94.8	14.5	15.9	352	0.045	Dipper

Increasing dietary sodium intake progressively elevated MAP across all conditions. However, the physiological effects of sodium loading depended strongly on the magnitude of tubular sodium transport rhythmicity. Under weak or absent tubular sodium rhythmicity, sodium loading produced higher mean MAP values and substantial redistribution of sodium excretion toward the nighttime period. In contrast, stronger tubular sodium rhythmicity attenuated BP elevation and preserved daytime-predominant natriuresis.

The influence of tubular sodium rhythmicity became increasingly important at higher sodium intake levels. Under the highest sodium loading condition, increasing tubular sodium rhythm amplitude progressively reduced the night/day urinary sodium excretion ratio from approximately 0.37–0.48 to approximately 0.045 while simultaneously enhancing nocturnal BP dipping to values exceeding 15%. Despite these changes in sodium handling and MAP amplitude, all completed simulations remained within the dipper range.

Together, these simulations suggest that intrinsic tubular sodium transport rhythmicity acts primarily as a stabilizing mechanism during sodium loading, promoting coordinated daytime sodium excretion and attenuating salt-induced BP elevation.

### Effect of tubular sodium transport phase alignment on BP dipping and natriuresis

**Fig 6:**
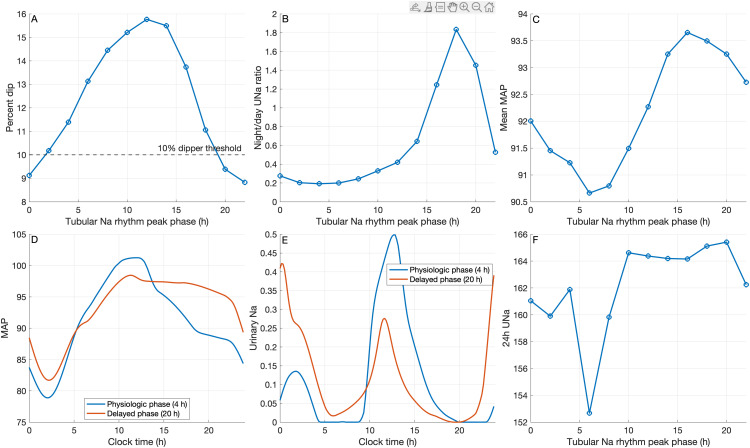
Effect of tubular sodium transport phase alignment on blood pressure dipping and natriuresis. Shifting the phase of the intrinsic tubular sodium transport circadian rhythm altered the temporal coordination between renal sodium handling and the sleep-wake cycle, producing substantial effects on BP regulation and sodium excretion timing. (A) Percent nocturnal BP dip varied strongly with tubular sodium transport phase, demonstrating sensitivity of the dipper phenotype to circadian phase alignment. The dashed horizontal line indicates the conventional 10% threshold separating dipper and non-dipper phenotypes. (B) The night/day urinary sodium excretion ratio increased markedly under delayed tubular sodium transport phases, indicating a shift toward greater nighttime sodium excretion. (C) Mean arterial pressure also varied with tubular sodium transport phase, with delayed phases associated with modest BP elevation. (D) Representative MAP waveforms for a physiologic tubular sodium transport phase (4 h) and a delayed phase (20 h). Delayed tubular sodium transport timing attenuated nocturnal BP dipping and elevated nighttime BP. (E) Representative urinary sodium excretion profiles illustrating redistribution of natriuresis toward the nighttime period under delayed tubular sodium transport timing. (F) Total 24-h urinary sodium excretion remained comparatively preserved across the phase sweep despite substantial changes in the temporal distribution of sodium excretion. Together, these simulations demonstrate that the relative timing among circadian renal regulatory pathways is a critical determinant of physiological BP dipping and coordinated natriuresis.

To investigate the importance of temporal coordination among circadian renal processes, the phase of the intrinsic tubular sodium transport rhythm was systematically varied while preserving all other circadian and sleep-wake modulation pathways (Fig 6, Table 6).

**Table 6:** Effect of tubular sodium transport phase on blood pressure dipping and sodium handling. Summary of simulations examining the effect of shifting the phase of the intrinsic tubular sodium transport circadian rhythm while preserving all other circadian and sleep-wake modulation pathways. Altering the timing of tubular sodium transport changed the coordination between renal sodium handling and the sleep-wake cycle, producing substantial effects on nocturnal BP dipping and the temporal distribution of sodium excretion. Misalignment of tubular sodium transport timing increased nighttime sodium excretion and attenuated physiological BP dipping, whereas favorable phase alignment promoted daytime-predominant natriuresis and preserved the dipper phenotype. These results demonstrate that the relative timing among circadian regulatory pathways is an important determinant of physiological BP regulation.

T_Na_ phase	MAP mean	MAP amp	MAP day	MAP night	Percent dip	UNa 24h	Night/day U_Na_	Status
0	92.0	8.77	94.9	86.3	9.12	161	0.28	Non-dipper
2	91.5	9.91	94.7	85.1	10.2	160	0.20	Dipper
4	91.2	11.2	94.9	84.1	11.4	162	0.19	Dipper
6	90.7	12.3	94.9	82.4	13.1	153	0.20	Dipper
8	90.8	12.8	95.5	81.7	14.4	160	0.24	Dipper
10	91.5	13.1	96.5	81.8	15.2	165	0.33	Dipper
12	92.3	13.3	97.5	82.1	15.8	164	0.42	Dipper
14	93.3	13.0	98.4	83.2	15.5	164	0.64	Dipper
16	93.7	11.5	98.2	84.7	13.7	164	1.2	Dipper
18	93.5	9.54	97.1	86.4	11.1	165	1.8	Dipper
20	93.3	8.38	96.3	87.2	9.39	165	1.5	Non-dipper
22	92.7	8.19	95.6	87.1	8.83	162	0.53	Non-dipper

Shifting the timing of tubular sodium transport produced marked effects on both BP dipping and the temporal distribution of sodium excretion. Phases aligned with the active period enhanced nocturnal BP dipping, with percent dip increasing from approximately 11% at the baseline phase to values exceeding 15% at phases near 10–14 h. In contrast, larger phase delays progressively attenuated BP dipping, with the model transitioning to a non-dipper phenotype at phases near 20–22 h.

Phase shifts also substantially altered natriuresis timing. Delayed tubular sodium transport phases redistributed sodium excretion toward the nighttime period, increasing the night/day urinary sodium excretion ratio from approximately 0.19 under baseline conditions to values exceeding 1 under strongly delayed phases. Representative urinary sodium excretion profiles demonstrated marked nocturnal natriuresis under delayed tubular sodium transport timing despite comparatively preserved total daily sodium excretion.

Thus, phase misalignment primarily altered the timing rather than the total magnitude of sodium excretion. Together, these simulations demonstrate that physiological BP dipping depends not only on the presence of circadian rhythms, but also on appropriate temporal alignment among renal circadian processes. Misalignment between tubular sodium transport timing and the sleep-wake cycle promotes nocturnal sodium excretion and predisposes the system toward non-dipping behavior.

### Salt-sensitive phenotypes amplify vulnerability to circadian phase misalignment

**Fig 7:**
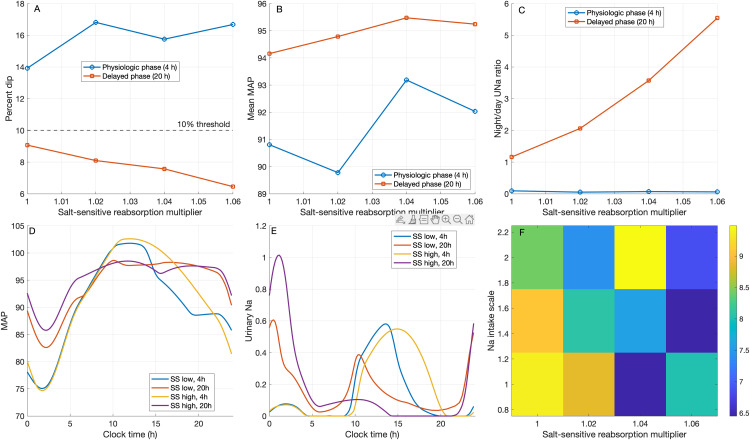
Salt-sensitive phenotypes amplify vulnerability to circadian phase misalignment. The effects of tubular sodium transport phase alignment were examined across progressively salt-sensitive phenotypes and dietary sodium loads. (A) Percent nocturnal BP dip as a function of the salt-sensitive reabsorption multiplier for physiologic tubular sodium transport timing (4 h phase) and delayed tubular sodium transport timing (20 h phase) under high sodium intake conditions (Na scale = 1.5). The dashed horizontal line indicates the conventional 10% threshold separating dipper and non-dipper phenotypes. Increasing salt sensitivity preserved or enhanced dipping under physiologic phase alignment but promoted non-dipping under delayed phase alignment. (B) Mean arterial pressure increased with salt sensitivity and was consistently higher under delayed tubular sodium transport timing. (C) The night/day urinary sodium excretion ratio increased markedly with salt sensitivity under delayed tubular sodium transport timing, indicating progressive redistribution of natriuresis toward the nighttime period. (D) Representative MAP waveforms comparing low salt sensitivity ($S_{\text{SS}}=\text{1}$) and high salt sensitivity ($S_{\text{SS}}=\text{1}.\text{0}\text{6}$) under physiologic tubular sodium transport timing (4 h phase) and delayed tubular sodium transport timing (20 h phase). (E) Representative urinary sodium excretion profiles for the same four conditions shown in panel D. Delayed tubular sodium transport timing produced substantial nighttime natriuresis, particularly in the high salt-sensitive phenotype. (F) Heatmap of percent nocturnal BP dipping across combinations of salt-sensitive reabsorption multiplier and dietary sodium intake under delayed tubular sodium transport timing (20 h phase). Together, these simulations demonstrate that salt-sensitive phenotypes are particularly vulnerable to circadian phase misalignment, which promotes nocturnal sodium excretion and non-dipping behavior during sodium loading.

To investigate how salt-sensitive renal physiology interacts with circadian timing, simulations were performed across combinations of dietary sodium loading, salt-sensitive tubular sodium reabsorption strength, and tubular sodium transport phase alignment (Fig 7, Table 7).

**Table 7:** Salt-sensitive tubular sodium reabsorption amplifies vulnerability to circadian phase misalignment. Summary of simulations examining the combined effects of salt-sensitive tubular sodium reabsorption, dietary sodium loading, and tubular sodium transport phase alignment on blood pressure regulation and sodium handling. Increasing the salt-sensitive tubular sodium reabsorption multiplier ($S_{SS}$) progressively enhanced the physiological consequences of delayed tubular sodium transport timing (T_Na_, 20 h phase), including attenuation of nocturnal BP dipping, elevation of mean arterial pressure (MAP, mmHg), and redistribution of urinary sodium excretion (UNa, mmol/day) toward the nighttime period. In contrast, physiologic tubular sodium transport timing (4 h phase) preserved robust BP dipping and daytime-predominant natriuresis even under substantial sodium loading. These simulations demonstrate that salt-sensitive phenotypes are particularly susceptible to circadian phase misalignment, which promotes non-dipping behavior and abnormal nocturnal sodium excretion. Estimated values derived from neighboring simulations are indicated where solver convergence was not achieved.

$S_{SS}$	Na intake x	T_Na_ phase	MAP mean	MAP amp	Percent dip	UNa 24h	Night/day ratio	Status
1	1	4	91.2	11.2	11.4	162	0.19	Dipper
1	1	20	93.3	8.38	9.39	165	1.5	Non-dipper
1	1.5	4	90.8	13.4	13.9	192	0.095	Dipper
1	1.5	20	94.2	8.01	9.07	248	1.2	Non-dipper
1	2	4	91.9	14.2	16.2	294	0.059	Dipper
1	2	20	95.1	7.72	8.43	330	1.2	Non-dipper
1.02	1	4	90.4	11.9	14.5	185	0.11	Dipper
1.02	1	20	93.7	8.23	8.85	165	2.0	Non-dipper
1.02	1.5	4	89.8	14.5	16.8	203	0.053	Dipper
1.02	1.5	20	94.8	7.80	8.10	249	2.1	Non-dipper
1.02	2	4	91.0	14.8	17.1	305	0.045	Dipper
1.02	2	20	95.9	7.35	7.43	331	2.3	Non-dipper
1.04	1	4	93.4	11.1	13.4	279	0.12	Dipper
1.04	1	20	94.1	7.90	8.21	166	3.1	Non-dipper
1.04	1.5	4	93.2	12.9	15.8	300	0.071	Dipper
1.04	1.5	20	95.5	7.47	7.57	249	3.6	Non-dipper
1.04	2	4	91.8	13.2	16.4	282	0.067	Dipper
1.04	2	20	95.7	8.74	9.48	317	0.84	Non-dipper
1.06	1	4	93.8	11.0	13.5	273	0.13	Dipper
1.06	1	20	94.7	7.83	8.03	167	5.3	Non-dipper
1.06	1.5	4	92.0	14.0	16.7	251	0.061	Dipper
1.06	1.5	20	95.2	6.37	6.45	256	5.5	Non-dipper
1.06	2	4	92.2	12.7	15.5	271	0.086	Dipper
1.06	2	20	94.3	7.85	6.86	387	2.4	Non-dipper

Under physiologic tubular sodium transport timing (4 h phase), the model preserved a robust dipper phenotype across a broad range of salt-sensitive conditions and dietary sodium loads. Despite enhanced sodium reabsorptive drive, daytime-predominant natriuresis remained preserved, with low night/day urinary sodium excretion ratios even under substantial sodium loading.

In contrast, delayed tubular sodium transport timing (20 h phase) consistently attenuated nocturnal BP dipping and redistributed sodium excretion toward the nighttime period. Under baseline salt sensitivity, delayed tubular sodium transport timing reduced nocturnal BP dipping below the conventional 10% dipper threshold, producing a non-dipper phenotype. The adverse effects of delayed tubular sodium transport timing became progressively amplified as salt sensitivity increased, with further reductions in nocturnal BP dipping, elevations in mean arterial pressure, and marked increases in the night/day urinary sodium excretion ratio. In several strongly salt-sensitive conditions, nighttime sodium excretion exceeded daytime sodium excretion by multiple-fold.

Representative MAP and urinary sodium excretion waveforms demonstrated that physiologic tubular sodium transport timing preserved robust nighttime BP reduction and daytime sodium excretion even under high sodium intake conditions, whereas delayed tubular sodium transport timing produced elevated nighttime BP together with pronounced nocturnal natriuresis. Heatmap analysis (Fig 7F) further demonstrated that the combination of sodium loading, enhanced tubular sodium reabsorption, and delayed tubular sodium transport timing strongly promoted non-dipping behavior.

Together, these simulations suggest that salt-sensitive phenotypes are particularly vulnerable to abnormalities in circadian renal timing. While physiologic tubular sodium transport timing partially buffered the hypertensive consequences of enhanced sodium reabsorption, delayed tubular sodium transport timing promoted nocturnal sodium excretion and destabilized physiological BP dipping during sodium loading.

### Relative contributions of endogenous circadian rhythms and sleep-wake modulation

**Fig 8:**
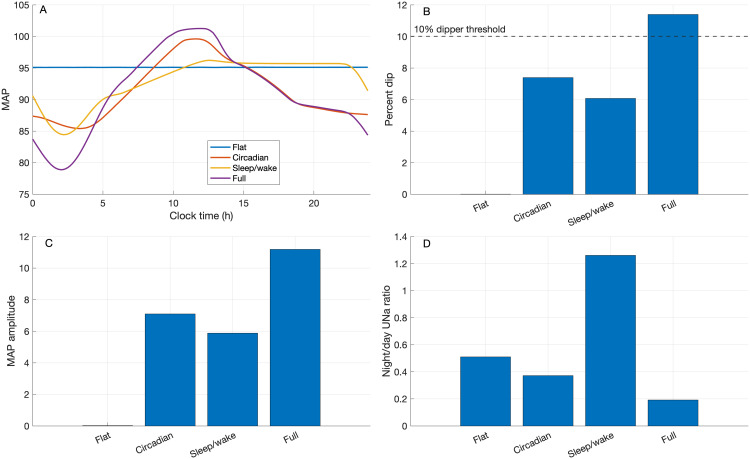
Relative contributions of endogenous circadian rhythms and sleep-wake modulation to physiological blood pressure dipping. Comparison of model behavior under four conditions: flat baseline (no circadian or sleep-wake modulation), endogenous circadian modulation only, sleep-wake modulation only, and the full integrated model containing both circadian and sleep-wake regulation. (A) Representative 24-h MAP waveforms. Circadian modulation alone generated substantial MAP rhythmicity but did not fully reproduce a physiological dipper phenotype, whereas sleep-wake modulation alone produced more modest BP oscillations. The full integrated model generated the largest and most physiologically realistic nocturnal BP dipping pattern. (B) Percent nocturnal BP dip for each condition. The dashed horizontal line indicates the conventional 10% threshold separating dipper and non-dipper phenotypes. Neither circadian modulation alone nor sleep-wake modulation alone was sufficient to produce a robust dipper phenotype, whereas the combined model exceeded the dipper threshold. (C) MAP amplitude under each condition. The full integrated model produced the greatest BP oscillatory amplitude. (D) Night/day urinary sodium excretion ratio. Circadian modulation alone promoted strong daytime-predominant natriuresis, whereas sleep-wake modulation alone shifted sodium excretion toward the nighttime period. Together, these simulations demonstrate that physiological BP dipping emerges from the interaction between endogenous circadian rhythms and behavioral sleep-wake modulation rather than either mechanism alone.

To distinguish the respective roles of endogenous circadian oscillators and behavioral sleep-wake modulation in generating physiological BP dipping, simulations were performed under four conditions: a flat baseline lacking both circadian and sleep-wake modulation, endogenous circadian modulation alone, sleep-wake modulation alone, and the full integrated model containing both regulatory systems (Fig 8, Table 8).

**Table 8:** Relative contributions of endogenous circadian rhythms and behavioral sleep-wake modulation to blood pressure dipping and sodium handling. Summary of simulations separating the effects of endogenous circadian rhythmicity and behavioral sleep-wake modulation on blood pressure regulation and natriuresis. Endogenous circadian modulation alone generated substantial MAP rhythmicity and promoted daytime-predominant sodium excretion but did not fully reproduce a physiological dipper phenotype. Sleep-wake modulation alone produced smaller BP oscillations and shifted sodium excretion toward the nighttime period. The full integrated model combining both circadian and sleep-wake regulation produced the most physiologically realistic dipper phenotype, including robust nocturnal BP reduction and coordinated daytime-predominant natriuresis. These simulations demonstrate that physiological BP dipping emerges from the interaction between endogenous circadian regulation and behavioral sleep-wake modulation rather than either mechanism alone.

Case	MAP mean	MAP amp	% dip	UNa 24h	Night/day UNa ratio	Status
Flat baseline	95.1	0.0185	0.0054	163	0.51	Non-dipper
Circadian only	91.4	7.10	7.39	160	0.37	Non-dipper
Sleep/wake only	92.9	5.88	6.07	164	1.3	Non-dipper
Full model	91.2	11.2	11.4	162	0.19	Dipper

In the absence of both circadian and sleep-wake modulation, the model produced an essentially flat BP profile with negligible MAP oscillatory amplitude and virtually absent nocturnal BP dipping. Urinary sodium excretion likewise lacked physiologic temporal organization, with a night/day urinary sodium excretion ratio near unity.

Endogenous circadian modulation alone generated substantial MAP rhythmicity but failed to fully reproduce a physiological dipper phenotype, with nocturnal BP dipping remaining below the conventional 10% threshold. Circadian modulation alone strongly promoted daytime-predominant natriuresis, driving the night/day urinary sodium excretion ratio toward near-zero values. This behavior reflected the absence of behavioral sleep-associated natriuresis relief in the circadian-only condition, resulting in near-complete suppression of nighttime sodium excretion. In contrast, sleep-wake modulation alone generated more modest BP oscillations and shifted sodium excretion toward the nighttime period, producing a night/day urinary sodium excretion ratio greater than unity.

The full integrated model combining endogenous circadian rhythms with sleep-wake modulation generated the most physiologically realistic phenotype, including robust nocturnal BP dipping, substantial MAP rhythmicity, and coordinated daytime-predominant natriuresis.

Together, these simulations demonstrate that physiological BP dipping emerges from the interaction between endogenous circadian timing mechanisms and behavioral sleep-wake modulation rather than either mechanism alone. Intrinsic circadian oscillators primarily organize cardiovascular and renal rhythmicity, whereas sleep-wake modulation contributes importantly to the emergence of a robust dipper phenotype and coordinated sodium handling.

### Local parameter sensitivity analysis

The principal model predictions were robust to modest parameter perturbations (Table 9). A ± 5% change in the amplitude of sleep–wake modulation produced only small changes in mean MAP (<0.2%), while MAP amplitude and percent nocturnal BP dip changed by less than approximately 3%. Similarly, varying the sleep-associated tubular sodium reabsorption relief term by ±5% had negligible effects on mean MAP (<0.1%), MAP amplitude (<1.5%), and percent BP dipping (<2%).

**Table 9:** Local parameter sensitivity analysis. Summary of simulations separating the effects of endogenous circadian rhythmicity and behavioral sleep-wake modulation on blood pressure regulation and natriuresis. Endogenous circadian modulation alone generated substantial MAP rhythmicity and promoted daytime-predominant sodium excretion but did not fully reproduce a physiological dipper phenotype. Sleep-wake modulation alone produced smaller BP oscillations and shifted sodium excretion toward the nighttime period. The full integrated model combining both circadian and sleep-wake regulation produced the most physiologically realistic dipper phenotype, including robust nocturnal BP reduction and coordinated daytime-predominant natriuresis. These simulations demonstrate that physiological BP dipping emerges from the interaction between endogenous circadian regulation and behavioral sleep-wake modulation rather than either mechanism alone.

Parameter	% change	%MAP mean	%MAP amp	% dip
Sleep/wake amp	−5	0.0405	−2.66	−1.92
Sleep/wake amp	+5	−0.148	2.83	3.14
Sleep Na relief	−5	0.0508	−1.38	0.324
Sleep Na relief	+5	0.0828	−1.489	−1.77
SS T_Na_ multiplier	−5	−0.0593	−15.4	−10.39
SS T_Na_ multiplier	+5	2.74	−1.92	17.9

In contrast, the model was more sensitive to the salt-sensitive tubular sodium reabsorption multiplier. A 5% reduction in this parameter produced modest decreases in MAP amplitude (approximately 15%) and nocturnal BP dipping (approximately 10%), with little effect on mean MAP. Conversely, a 5% increase elevated mean MAP by approximately 2.7% and increased nocturnal BP dipping by approximately 18%, while producing only a small reduction in MAP amplitude.

Overall, these results indicate that the principal conclusions of the study are insensitive to modest uncertainty in the parameters governing sleep–wake modulation and sleep-associated tubular sodium handling. Greater sensitivity to the salt-sensitive tubular sodium reabsorption multiplier reflects the central role of renal sodium reabsorption in determining the long-term operating point of the pressure–natriuresis system. Importantly, however, the qualitative behaviors identified throughout this study—including the dependence of BP dipping on circadian timing and temporal coordination—were preserved across all parameter perturbations.

## Discussion

In this study, we developed an integrated circadian-sleep/wake model of BP regulation that couples rhythmic neural, vascular, endocrine, and renal sodium transport processes to investigate the physiological determinants of nocturnal BP dipping and coordinated natriuresis. The simulations reproduced a physiologically realistic dipper phenotype and generated several mechanistic insights regarding the origins of non-dipping behavior. In particular, the results suggest that physiological BP dipping is an emergent systems-level property arising from coordinated interactions among vascular rhythmicity, sleep-associated autonomic modulation, and temporally organized renal sodium handling rather than from a single dominant oscillator. This systems-level interpretation is consistent with growing evidence that non-dipping reflects multifactorial dysregulation involving renal sodium handling, circadian timing, autonomic regulation, and vascular function [3,13].

One of the central findings of this study is that vascular rhythmicity and behavioral sleep-wake modulation emerged as dominant determinants of nocturnal BP dipping, whereas rhythmicity within the renin-angiotensin-aldosterone system exerted comparatively modest effects under baseline conditions. Removal of circadian vascular modulation substantially attenuated dipping and produced a non-dipper phenotype, consistent with human and experimental studies showing circadian variation in endothelial function, vascular function, and vasoconstrictor responses [37–39]. Similarly, elimination of sleep-associated suppression of sympathetic and vascular drive impaired nocturnal BP reduction despite preservation of endogenous circadian oscillators. Experimental studies have likewise demonstrated reduced sympathetic activity, lower vascular tone, and reduced arterial pressure during normal sleep [10,31]. Together, these results support the concept that physiological dipping depends not only on intrinsic circadian timing but also on appropriate behavioral and autonomic transitions associated with sleep.

A second important finding is that intrinsic tubular sodium transport rhythmicity primarily regulated the temporal distribution of sodium excretion rather than acting as a dominant generator of BP dipping itself. Across multiple simulation studies, reduction of tubular sodium transport rhythmicity produced relatively modest effects on the magnitude of nocturnal BP dipping while substantially redistributing sodium excretion toward the nighttime period. Interestingly, total 24-h urinary sodium excretion remained comparatively preserved despite large changes in excretion timing. These simulations therefore suggest that circadian renal transport rhythms function primarily as temporal coordinators that align natriuresis with behavioral and cardiovascular rhythms. This interpretation is consistent with experimental evidence demonstrating circadian regulation of nephron sodium transporters and renal epithelial clock mechanisms [7,8]. The present results also extend classical pressure-natriuresis concepts by emphasizing not only the quantity of sodium excreted, but also the timing of sodium excretion across the circadian cycle. Clinical and experimental studies have similarly linked impaired daytime natriuresis with nocturnal hypertension and non-dipping phenotypes [11,12].

The simulations examining reduced tubular sodium transport rhythmicity should be interpreted primarily as a mechanistic perturbation rather than a model of a specific disease state. To our knowledge, selective loss of tubular sodium transport rhythmicity has not been demonstrated experimentally. Instead, renal circadian dysfunction likely occurs together with broader disturbances in circadian organization, such as those accompanying aging, shift work, sleep disorders, chronic kidney disease, or metabolic disease. By selectively reducing tubular transport rhythmicity while preserving other regulatory pathways, the model isolates the specific contribution of this mechanism to blood pressure regulation and natriuresis.

The phase-shift simulations further demonstrated that temporal coordination among physiological rhythms may be as important as rhythm amplitude itself. Delayed tubular sodium transport timing alone was sufficient to convert the model from a dipper to a non-dipper phenotype despite preservation of rhythmic oscillations in the remaining physiological systems. Thus, non-dipping behavior may emerge not only from loss of rhythmicity, but also from mistimed interactions among otherwise intact oscillators. This finding has potential implications for conditions associated with circadian disruption, including shift work, sleep disorders, aging, chronic kidney disease, obesity, and metabolic disease, all of which are associated with increased prevalence of non-dipping hypertension [3]. Experimental and clinical studies have increasingly linked circadian misalignment with adverse cardiovascular and renal outcomes, although the physiological mechanisms remain incompletely understood [40]. The present simulations suggest that impaired temporal alignment between renal sodium handling and the sleep-wake cycle may represent one such mechanism.

The interaction between salt sensitivity and circadian timing produced particularly striking behavior. Clinically, both salt-sensitive hypertension and high dietary sodium intake are associated with increased prevalence of non-dipping phenotypes and abnormal nocturnal sodium handling [3,11,32]. In the present simulations, however, sodium loading and enhanced tubular sodium reabsorption alone did not necessarily abolish physiological dipping when tubular sodium transport timing remained appropriately aligned with the active period. Under these conditions, coordinated daytime natriuresis was largely preserved despite elevated sodium retention and increased BP. In contrast, delayed tubular sodium transport timing markedly amplified the adverse effects of sodium loading and salt-sensitive sodium retention, promoting nocturnal natriuresis, elevated nighttime BP, and non-dipping behavior. These results suggest that sodium loading may predispose the system toward non-dipping primarily when temporal coordination of sodium excretion becomes impaired. More broadly, the simulations support the concept that non-dipping may reflect a failure of coordinated daytime sodium excretion, requiring BP elevation during the sleep period to maintain sodium balance, as proposed in kidney-centered interpretations of non-dipping hypertension [13].

The comparison between endogenous circadian rhythms and behavioral sleep-wake modulation further emphasized the importance of systems-level coordination. Circadian modulation alone generated substantial MAP rhythmicity and strongly daytime-predominant natriuresis but did not fully reproduce a physiological dipper phenotype. Conversely, sleep-wake modulation alone generated modest BP oscillations but poorly coordinated sodium excretion timing. Only the combined model reproduced robust nocturnal BP dipping together with physiologically coordinated natriuresis. These findings are consistent with experimental studies demonstrating that endogenous circadian oscillators and behavioral sleep-state transitions independently contribute to cardiovascular rhythmicity [6].

The present study also generates several experimentally testable hypotheses. First, the simulations predict that altered phase relationships among renal transport rhythms may contribute importantly to non-dipping phenotypes, even in the absence of major reductions in rhythm amplitude. Experimental characterization of nephron transporter timing in non-dipper models may therefore provide important mechanistic insight. Second, the simulations suggest that restoration of temporal coordination among renal sodium transport pathways may represent a potential therapeutic strategy for non-dipping hypertension. More generally, the model supports the emerging concept that chronobiological organization may influence renal sodium handling and BP regulation independently of changes in average transport magnitude. These predictions are broadly consistent with growing interest in chronotherapy and circadian-targeted cardiovascular interventions, although the optimal timing of antihypertensive therapy in clinical practice remains an area of active investigation [26,41,42]. Although the model examined phase shifts of tubular sodium transport in isolation to establish mechanistic causality, such isolated perturbations are unlikely to occur in vivo. Peripheral renal clocks are entrained by systemic cues, including feeding time, hormonal signals, and autonomic input, and circadian disruption would therefore be expected to alter multiple physiological rhythms simultaneously. Mistimed feeding, shift work, or other circadian perturbations may thus induce more complex patterns of temporal misalignment than those represented here. Nevertheless, isolating tubular sodium transport timing demonstrates that disruption of this pathway alone is sufficient to impair coordinated natriuresis and promote non-dipping behavior, identifying it as a potentially important mechanistic contributor within the broader circadian network.

Recent studies by Ueda et al. [43] demonstrated that chronic intermittent hypoxia induces a non-dipping phenotype that is reversible by dietary sodium restriction and is accompanied by increased renal sodium transporter activity. Although intermittent hypoxia was not explicitly modeled here, our simulations suggest a mechanistic interpretation. Increased transporter activity alone produced relatively modest changes when normal circadian timing was preserved, whereas the combination of enhanced sodium reabsorption with circadian phase misalignment markedly promoted nocturnal sodium retention and non-dipping behavior. These results raise the possibility that intermittent hypoxia alters not only the magnitude of renal sodium transport but also its temporal coordination with the sleep-wake cycle. Furthermore, the present model suggests that the intermittent hypoxia phenotype is unlikely to be explained solely by increased sodium transporter activity. Rather, it may arise from the combination of enhanced tubular sodium reabsorption together with altered circadian timing and sleep-associated regulation of renal sodium handling.

The present results may also have implications for chronopharmacology and circadian disruption. The simulations suggest that the timing of renal sodium transport relative to behavioral sleep-wake cycles substantially influences BP dipping and natriuresis, raising the possibility that time-of-day-dependent therapeutic interventions could improve non-dipping phenotypes [26]. More broadly, the phase-misalignment simulations provide a potential mechanistic framework linking circadian disruption associated with shift work, irregular sleep schedules, and sleep disorders to non-dipping hypertension and altered nocturnal sodium handling [6,40]. These findings support the emerging concept that restoration of appropriate temporal coordination among cardiovascular and renal rhythms may represent an important therapeutic target.

Several limitations should be acknowledged. Circadian rhythms were imposed phenomenologically using prescribed modulation functions rather than generated through explicit molecular clock network dynamics. Thus, the present framework was designed to investigate systems-level physiological consequences of coordinated rhythmic regulation rather than intracellular clock gene mechanisms. In addition, the model does not explicitly represent feeding rhythms, physical activity variation, sleep-stage structure, sex differences, or nephron segment-specific molecular clock networks. The present study also focused primarily on short-term circadian BP regulation and did not incorporate long-term cardiovascular remodeling or structural renal adaptation. Additionally, the present simulations were performed using the female parameterization of the underlying renal transport model. Although the principal systems-level mechanisms identified here are expected to generalize qualitatively across sexes, quantitative sex differences in renal transporter expression, sodium handling, and BP regulation may alter the relative magnitudes of circadian and salt-sensitive responses. Future studies should therefore examine sex-specific determinants of circadian BP dipping and non-dipping behavior.

In conclusion, the simulations support the concept that physiological BP dipping reflects successful temporal coordination among cardiovascular, renal, and behavioral regulatory systems. Non-dipping behavior may therefore emerge not only from impaired rhythmicity, but also from misalignment among otherwise preserved physiological oscillators. The model further suggests that circadian coordination of renal sodium handling becomes increasingly important during sodium loading and in salt-sensitive phenotypes, highlighting the potential importance of temporal renal physiology in the pathogenesis of non-dipping hypertension.Top of FormBottom of Form
